# Obstructive Lung Disease among Patients Performing Spirometry in a Tertiary Care Centre: A Descriptive Cross-sectional Study

**DOI:** 10.31729/jnma.7455

**Published:** 2022-09-30

**Authors:** Prinsa Shrestha, Subash Pant

**Affiliations:** 1 Kathmandu Medical College and Teaching Hospital, Sinamangal, Kathmandu, Nepal; 2 Department of Internal Medicine, Kathmandu Medical College and Teaching Hospital, Sinamangal, Kathmandu, Nepal

**Keywords:** *chronic obstructive pulmonary disease*, *obstructive lung diseases*, *prevalence*, *spirometry*

## Abstract

**Introduction:**

Obstructive lung disease is a leading cause of morbidity and mortality worldwide which causes economic and social burdens. Spirometry is a standard test for screening and evaluating patients with symptoms of chronic respiratory diseases and is the investigation of choice for identifying airflow obstruction. Chronic obstructive lung disease prevalence, mortality, and morbidity vary across different countries. The study aimed to find out the prevalence of obstructive lung disease among the patients performing spirometry in a tertiary care centre.

**Methods:**

A descriptive cross-sectional study was conducted in the pulmonary function test clinic of the Department of Internal Medicine in a tertiary care hospital between 1 October 2021 and 31 March 2022 from hospital records. Ethical approval was taken from the Institutional Review Committee (Reference number: 200320203). Convenience sampling was done. Point estimate and 95% Confidence Interval were calculated.

**Results:**

Among 401 patients, obstructive lung disease was present in 173 (43.14%) (38.29-47.99, 95% Confidence Interval). The mean age was 55.78±18.54 years. The most common symptom for referral was dyspnea seen in 151 (87.30%).

**Conclusions:**

The prevalence of obstructive lung disease in our study was higher compared to other studies from the similar setting. Spirometry should be used more frequently for diagnosis and to stratify patients for appropriate treatment.

## INTRODUCTION

Obstructive lung disease is a leading cause of morbidity and mortality worldwide. It results in an economic and social burden that is both substantial and increasing.^1,2^ It includes chronic obstructive pulmonary disease (COPD) and bronchial asthma.

Spirometry is a standard test for screening and evaluation of patients with symptoms of chronic respiratory diseases like dyspnea and cough.^3^ It is a simple test with easy availability, low cost, ease of performance, good reliability, and acceptability of the results. Therefore, spirometry is the investigation of choice for the identification of airflow obstruction.^2-6^ COPD prevalence, mortality, and morbidity vary across countries, and across different groups within countries. So far, there are only a few studies conducted in Nepal to find the prevalence of obstructive lung disease.

This study aimed to find the prevalence of obstructive lung disease among the patients performing spirometry in a tertiary care centre.

## METHODS

A descriptive cross-sectional study was conducted in Kathmandu Medical College and Teaching Hospital (KMCTH), Kathmandu, Nepal from 1 October 2021 to 31 March 2022. Ethical approval was taken from the Institutional Review Committee of KMCTH (Reference number: 200320203). The data was collected from the medical record section. The study population was patients performing spirometry in the pulmonary function test (PFT) clinic of KMCTH. Patients of age >18 years who visited the PFT clinic during the study period who fulfilled the ATS (American Thoracic Society)/ERS (European Respiratory Society) criteria of spirometry were included in the study.^3^ Participants with a history of respiratory infection or exacerbation of COPD or asthma within 6 weeks before the recruitment were excluded from the study. Convenience sampling was done. The sample size was calculated using the formula:


$$\begin{array}{ll} \text{n} & ={\text{Z}}^{2}\times \frac{\text{p}\times \text{q}}{{\text{e}}^{\text{2}}}\\ & =1.96^{2}\times \frac{0.5\times 0.5}{0.05^{2}}\\ & =385 \end{array}$$

Where,

n= required sample size
Z= 1.96 at 95% Confidence Interval (CI)
p= prevalence taken as 50% for maximum sample size calculation
q= 1-p
e= margin of error, 5%

The calculated sample size was 385. However, 401 participants were taken.

All the spirometry tests were guided by a single trained technician, and interpretation of all the spirometry reports was done by a single doctor. Obstructive lung disease is defined as post-bronchodilator Forced Expiratory Volume in the first second and Forced Vital Capacity (FEV1/FVC) ratio <0.70.^1,2^ COPD is defined as FEV1/FVC ratio less than 70% predicted without significant bronchodilation, defined as less than 12% or 200 ml increase in forced expiratory volume in the first second (FEV1) half an hour after 400 mcg of salbutamol inhalation in patients with appropriate symptoms (dyspnea, cough, sputum production) and significant exposure to noxious stimuli.^1,2^

In patients with FEV1/FVC<0.70, the degree of obstruction is categorised as mild, moderate, severe and very severe. Patients were categorised as mild if FEV1≥80% of the predicted value; moderate if FEV1 is 50-79% of the predicted values; severe if FEV1 is 30-49% of the predicted values; and very severe if FEV1<30% of the predicted values. These categories define GOLD stage I-IV disease respectively using the Global Initiative for Obstructive Lung Disease (GOLD) criteria.^2^ Using spirometry, bronchial asthma is defined as FEV1/FVC<70% with significant bronchodilation defined as ≥12% and ≥200 ml increase in FEV1 half an hour after 400 mcg of salbutamol inhalation or corticosteroid treatment.^7,8^ Peak expiratory flow (PEF) was also measured. Data were entered and analysed in IBM SPSS Statistics 24.0. Point estimate and 95% CI were calculated.

## RESULTS

Out of 401 patients, 173 (43.14%) (38.29-47.99, 95% CI) had obstructive lung disease. There were 76 (43.90%) males and 97 (56.10%) females. The age range of the patients was 18-88 years with a mean age of 55.78±18.54 years. The mean Body Mass Index of the patients was 21.73±4.25 kg/m^2^. Among the patients, 169 (97.70%) were of South East Asian ethnicity, and four (2.30%) were Caucasian. The occupation of the majority of the patients was household 76 (43.93%) (Table 1).

**Table 1:** Occupation of patients with obstructive lung disease (n= 173).

Occupation	n (%)
Household	76 (43.93)
Farmer	61 (35.26)
Service	16 (9.20)
Student	15 (8.70)
Business	5 (2.90)

A total of 67 (38.73%) patients with obstructive lung disease were current smokers, 63 (36.40%) were former smokers and 43 (24.90%) were non-smokers. The most common symptom for referral in these patients was dyspnea which was present in 151 (87.30%) patients followed by a chronic cough which was present in 95 (54.90%) patients (Table 2).

**Table 2:** Referral symptoms for spirometry in patients with obstructive lung disease (n= 173).

Symptoms	n (%)
Dyspnea	151 (87.30)
Chronic cough	95 (54.90)
Wheeze	66 (38.20)
Pre-operative evaluation	12 (6.90)

The mean FEV1/FVC was 0.59±0.13 in pre-bronchodilator spirometry in patients with obstructive ung disease (Table 3).

**Table 3:** Pre-bronchodilator spirometric parameters of patients with obstructive lung disease (n= 173).

Parameters	Mean±SD
FEV1 (l)	1.52±1.52
FEV1 (% predicted)	57.42±20.4
FVC (l)	2.16±0.77
FVC (% predicted)	75.75±19.01
FEV1/FVC	0.59±0.13
FEV1/FVC (% predicted)	73.46±14.83
PEF (l/min)	2.96±1.52

The mean FEV1/FVC was 0.58±0.14 in post-bronchodilator spirometry (Table 4).

**Table 4:** Post-bronchodilator spirometric parameters of patients with obstructive lung disease (n= 173).

Parameters	Mean±SD
FEV1 (l)	1.33±0.57
FEV1 (% predicted)	58.76±18.56
FVC (l)	2.27±0.79
FVC (% predicted)	78.76±16.75
FEV1/FVC	0.58±0.14
FEV1/FVC (% predicted)	72.67±16.22
PEF (l/min)	2.97±1.53

Among the patients with obstructive lung disease, significant reversibility was seen in 31 (17.90%) patients, while reversibility was insignificant in 142 (82.08%) patients who were categorized as COPD patients. Most of the COPD patients were in GOLD stages II and III (Table 5).

**Table 5:** GOLD staging of the patients with obstructive lung disease (n= 173).

GOLD stage Severity	n (%)
I Mild	33 (19.08)
II Moderate	73 (42.20)
III Severe	44 (25.40)
IV Very severe	23 (13.30)

## DISCUSSION

In this study, we have found the prevalence of obstructive lung disease among patients performing spirometry in a tertiary care centre to be 173 (43.14%) which is very high compared to the published literature.^7-14^ A systematic review reported the prevalence of obstructive lung disease ranging from 0.23-18.3%.^7^ The prevalence of COPD from a study conducted in Copenhagen was 3.7%,^8^ in Korea was 7.8%,^9^ in the UK was 26.4%^10^ in two different studies conducted in Nepal was 42%^11^ and 31.4%.^12^ This vast difference may be due to the difference in the way of recruitment of the patients. We have found the prevalence only among the patients who were referred for spirometry rather than including all the patients who visited the outpatient department. Since mostly symptomatic patients with high suspicion of respiratory pathology are referred for spirometry, the prevalence of obstructive lung disease was found to be higher in our study.

The most common symptoms for referral were dyspnea and chronic cough which was comparable to another study conducted in Nepal in which both dyspnea and chronic cough were present in 54.2%^12^ of patients. The occupation of the majority of our patients was household (43.93%) followed by farming (35.26%) which was similar to a previous study conducted in Nepal.^12^ These people usually have a history of exposure to domestic smoke in the form of firewood, and also belong to low economic status. Furthermore, most of the patients were either current smokers (38.73%) or former smokers (36.4%). This suggests that obstructive lung disease is more prevalent among low economic status and those exposed to firewood or cigarette smoke.^13,14^

The majority of the COPD patients were in GOLD stages II (42.2%) and III (25.4%) which was similar to another study conducted in Nepal in which 42.2% of the patients were in GOLD II and 28.3% were in GOLD III.^12^ This highlights the fact that patients only develop symptoms after there has been a significant loss of lung function, often to 50 or 60% of the predicted value. Due to this, patients present to the health care centre late in the course of the disease. Thus, early detection of this disease with spirometry is essential to start appropriate treatment, to help decrease the associated morbidity and mortality.

Since our study was a single-centred study, with convenience sampling, the results cannot be generalised to the whole population. Another limitation of our study is that cut-off values of FEV1/FVC may vary according to age group and gender. Since participants included in our study are of various age groups, fixed cut-off values of FEV1/FVC <0.70 defining obstructive lung disease may not hold true for all the participants.

## CONCLUSIONS

The prevalence of obstructive lung disease in our study was found to be higher compared to other studies from similar settings. It is recommended to conduct a national multi-centre study to find the prevalence of obstructive lung disease in the Nepalese population. Spirometry could be important in the early evaluation, diagnosis, and subsequent monitoring of respiratory diseases mainly associated with airway obstruction.
